# TEAD inhibitor induced switch in energy metabolism drives human podocyte injury in vitro

**DOI:** 10.1007/s00204-026-04497-7

**Published:** 2026-07-06

**Authors:** Alexander Zielinski, Julia Hauptstein, Bernd Baier, Peter Schaarschmidt, Natalie Ketter, Daniel R. Dietrich, Florian Meier

**Affiliations:** 1 Human & Environmental Toxicology, University of Konstanz, Konstanz, Germany; 2 Boehringer Ingelheim Pharma GmbH & Co.KG, Global Non-clinical Safety Science Germany, Biberach an der Riss, Germany

**Keywords:** Kidney, Proteinuria, TEAD, Podocytes, Palmitic acid, Mitochondrial function

## Abstract

Early detection and understanding of the potential adverse effects of a drug candidate is of utmost importance to reduce the attrition rate during drug development while delivering safe medications to patients. The kidney is one of the most frequently challenged organs involved in drug-induced toxicity, including general glomerulopathy and podocyte- specific cytotoxicity with ensuing overt proteinuria. Whilst TEAD inhibitors address the need for treatment of mesotheliomas and other tumours unaffected by approved drugs, animal testing and clinical trials provide evidence of renal toxicity induced by this novel class of drugs. Here we present an in vitro test system consisting of immortalized human podocytes PODO/TERT256 to characterize the potential adverse effects of TEAD inhibitors. We demonstrate that TEAD inhibitors K-975, IK-930 and VT-107 affect the podocyte monolayer in a wide dose range (33.3–100 µM), disrupt the cellular vesicle system, and subsequently induce cell death, especially after exposure to pan-TEAD inhibitors (VT-107 and K-975). Of note, low micromolar concentrations of TEAD inhibitors lowered mitochondrial oxidative phosphorylation directly after exposure which was confirmed by reduced basal ATP levels after 3 d of exposure. In summary, the in vitro human podocyte model presented can characterize glomerular filtration functionality decline subsequent to TEAD inhibitor exposure and thus represents a promising model for early detection and prediction of potential drug related glomerulopathy in humans.

## Introduction

**Fig. 1:**
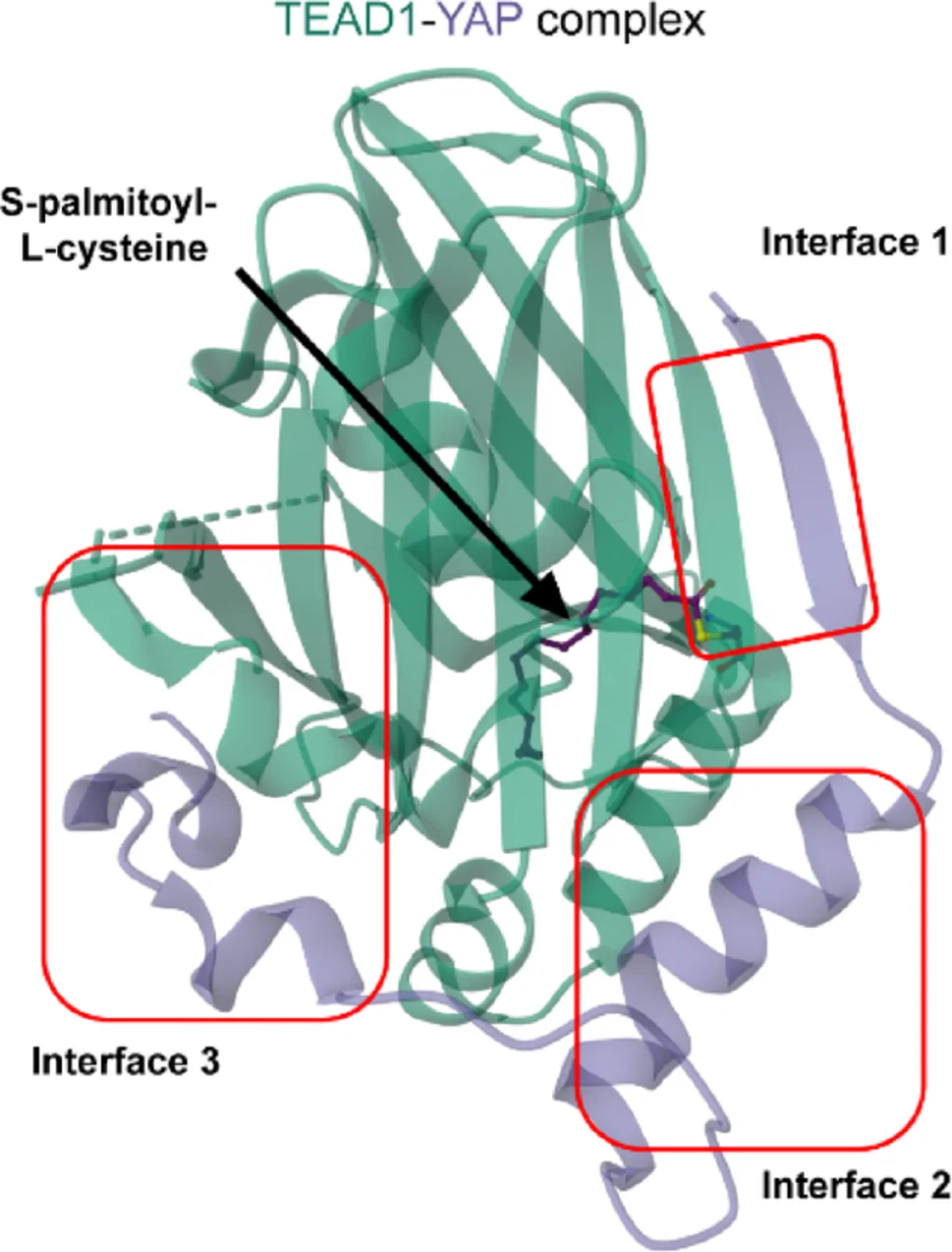
Crystal structure of human TEAD1-YAP complex including the S-palmitoyl-L-cysteine at position 344 occupying the central hydrophobic pocket (PDB ID: 3KYS). The original structure has been published by (Li et al. 2010) on RCSB.org (Berman et al. 2000). The figure was created using Mol* Viewer (Sehnal et al. 2021) and is based on Pobbati et al. (Pobbati et al. 2023)

TEAD (yes-associated protein (YAP)/transcriptional enhancer activator domain) proteins are the central part of the Hippo pathway, as they combine, via binding of their cofactors YAP (Yes-associated protein) and/or TAZ (transcriptional co-activator with PDZ-binding motif), to an active transcription factor complex (Noland et al. 2016; Sun et al. 2022). The amplification as well as genetic alteration of TEAD (TEA/ATTS domain) genes and thus altered TEAD transcription-factor-complex deregulation is associated with various cancers, including lung, liver, skin, breast and colon cancers (Paul et al. 2025). Therefore, the inhibition of the TEAD function or abrogating the formation of the transcription-factor-complex appears a promising target to prevent proliferation, epithelial to mesenchymal transition and metastasis of cancer cells. The protein structure of the TEAD proteins, which consist of four members (TEAD1-4), present with a hydrophobic pocket and a number of interfaces that bear promise to being druggable (Luo et al. 2022; Noland et al. 2016). Key appears to be the central hydrophobic binding pocket containing a cysteine residue in the YAP-binding domain, which can be S-palmitoylated, and is conserved across all four TEAD family members (Pobbati et al. 2015). However, in vitro data also suggest that TEAD interaction and consequently transcription, can occur independently of the palmitoylation status (Sun et al. 2022). In addition to the hydrophobic pocket (Fig. 1), there are 3 interfaces for the interaction with YAP/TAZ, with interface 2 and 3 as promising druggable targets for potential protein-protein interaction disruptors (PPIDs). Accordingly, a number of compounds are currently undergoing preclinical and/or phase I testing (Luo et al. 2022). Among the most advanced compounds is IAG933 (Novartis), which is deemed to interact with the interface 3 (Chapeau et al. 2024). In contrast, IK-930 (Ikena Oncology), VT3989 and VT-107 (Vivace Therapeutics) and K-975 (Kyowa Kirin Co. Ltd) all address the central hydrophobic pocket, whereby IK-930 preferable inhibits TEAD1, while VT3989, V-T107 and K-975 appear to act as pan-TEAD inhibitors (Kaneda et al. 2020; Tang et al. 2021; Tolcher et al. 2022; Young et al. 2023).

Of the latter four compounds, IK-930, VT3989 and K-975 were tested in preclinical trials, primarily in rats, and in some cases in non-human primates, and all demonstrated renal adverse events suggestive of a glomerulopathy e.g. proteinuria to a variable and, in some cases, reversible degree (Young et al. 2023). Ensuing histopathological and electron microscopic analyses suggested that proteinuria did not result from overt podocyte toxicity but rather from podocyte foot process effacement and cytoskeletal rearrangement. The latter suggesting that these adverse effects were reversible upon cessation of exposure (Otsuki et al. 2024). A phase I trial with VT 3989 reported reversible albuminuria (53.5% of patients; Grade 1–3), which was the most common adverse event (Yap et al. 2023a). Similarly, Ikena reported for IK-930 albuminuria in the Phase 1 trial, however, with a lower incidence (12%) and Grading (1–2) (Tolcher et al. 2022). These findings, alongside the described phenotypes of YAP or TAZ or TEAD KO/conditional KO in mice (Kakiuchi-Kiyota et al. 2019), pointed to the kidney and particularly the glomerulus being a highly susceptible tissue in conjunction with Hippo pathway modulation. Indeed, proteinuria would typically evolve when the fenestrae of the glomerular capillary endothelium become enlarged, which occurs, amongst other factors, concomitant or secondary to retreating podocyte foot processes and/or podocyte effacement (Kopp et al. 2020; Shankland 2006). The analyses of preclinical as well as clinical phase I trials, however, also demonstrated that proteinuria was not consistently detectable in all animals or patients. Moreover, proteinuria was present in affected animals and patients to a varying degree, thereby suggesting that preclinical trials could potentially underestimate or miss the adverse effect of podocyte foot process effacement and cytoskeletal rearrangement by merely using proteinuria as a biomarker of adversity. Therefore, a human in vitro model to predict these changes at an early phase of drug development would be preferable. For in vitro testing of human podocytes, characterization of basic traits and functionality was deemed prerequisite (Pavenstadt et al. 2003). Therefore, focus was laid on PODO/TERT256 cells for which a thorough characterization of the morphology and functionality was already available (Schlichenmaier et al. 2024). Accordingly, PODO/TERT256 were employed to determine the in vitro effect of IK-930, VT-107 and K-975 on human podocyte viability, morphology, functionality (particle size selectivity, impedance) and basic metabolism (Seahorse and ATP levels). In view of the transient responses of cancer cells to TEAD inhibitors reported (Sun et al. 2022), co-exposure experiments with palmitic acid were carried out with the aim to potentially distinguish target from off-target effects of the TEAD inhibitors tested, thereby allowing for an improved early detection and prediction of potential drug related glomerulopathy in human patients.

## Methods

### Cell Culture

PODO/TERT256 cells were obtained from Evercyte GmbH, Austria (Cat# CHT- 033–0256). PODO/TERT256 were cultured in a 1:1 mix of DMEM no glucose (Cat# 11,966,025, Thermo Fisher) and Ham’s F12 nutrient mix (Cat# 11,765,054, Thermo Fisher) supplemented with ITS-G solution (Cat# 41,400,045, Thermo Fisher), 2 mM GlutaMax (Cat# 35,050,061, Thermo Fisher), 100 U/ml Penicillin/Streptomycin (Thermo Scientific) and 1% FCS (Cat# FBS.GP.0500 Bio&Sell). Cell culture plastic was pre-coated with 50 µg/ml human collagen I (Cat# 5007, Cell Systems) in PBS for 30 min at 37 °C to facilitate cell attachment. Cells were cultured at 5% CO_2_, 37 °C, 95% humidity with medium renewal every second to third d. Morphology was assessed by microscopy (EVOS M5000, Invitrogen). Cells were sub-cultured (split) once per week and cell numbers counted using a Neubauer counting chamber.

### Cell viability and cell death assay

Cells were treated with IK-930, VT-107 and K-975 (all TEAD inhibitors employed were obtained from MedChemExpress) in the presence of 0.25% dimethyl sulfoxide (DMSO, Sigma-Aldrich) or with 0.25% DMSO (solvent control). To quantify cell viability and cell death supernatant was collected post-treatment and exchanged for fresh medium containing 0.11 mg/ml resazurin sodium salt (Cat# R7071, Sigma-Aldrich). After incubation at 37 °C for 90 min the resazurin solution was collected in a half area black well plate (Cat# 675076, Greiner Bio-One) and fluorescence (EX = 500 nm, EM = 500 nm) was measured with a Tecan M200Pro microplate reader (Tecan). Values obtained for dead cells (0.1% Triton X-100 treated), see below, were subtracted from all other values and data were expressed as percentage of control cells. Cell death was analysed separately by quantification of lactate dehydrogenase (LDH; Sigma-Aldrich) activity in previously collected supernatant and cell lysate. For this, cells were lysed in 0.1% Triton X-100/phosphate-buffered saline (PBS). Leakage of LDH into the cell culture medium was analysed enzymatically based on the reduction of NADH to NAD at 37 °C and the reduction in absorbance at 340 nm. LDH leakage was expressed as percentage of LDH activity in treatment supernatant divided by the combined activity in supernatant and lysate (Schildknecht et al. 2009).

### NanoLive holotomography

PODO/TERT256 cells were cultured for 2 or 7 d in collagen coated µ-Dish 35 mm (Cat.# 81156, Ibidi). Cells were treated with IK-930, VT-107, K-975 or 0.25% DMSO before being inserted in the Nanolive Cell Explorer CX-A incubation chamber (NanoLive) at the Bioimaging Centre of the University of Konstanz. After manual optimization of the focus holotomographic pictures were taken every 30 min for 24 h. Images were then adjusted for display using ImageJ (Schneider et al. 2012).

### Permeability assay

For measuring cell monolayer permeability (apparent permeability), PODO/TERT256 were cultured for 14 d on 24-well transwell inserts with a growth area of 0.3 cm^2^ and 0.4 μm pores (Cat.# 83.3932.041, Sarstedt). First, 0.5 mg/ml of fluorescently labelled dextrans (FITC-Dextran, MW = 70 kDa (Cat.# 90,718, Sigma); TRITC-Dextran, MW = 20 kDa (Cat.#73766, Sigma) were added to the basolateral chamber and incubated at 37 °C and 5% CO_2_. After 4 h 50 µl were transferred from the upper well into a black half area plate. Fluorescence was measured using a microplate reader (M200Pro, Tecan) at Ex = 493 nm/Em = 520 nm (FITC) and Ex = 550 nm/Em = 577 nm (TRIC). Amount of dextran was calculated using point-to-point regression and a dextran standard curve (0–0.6 mg/ml). The apparent permeability coefficient of the layer was calculated according to the following formula as published in (Hubatsch et al. 2007):

$$\:apparent\:permeability\:\left(\frac{cm}{sec}\right)=\:\frac{V\left(ml\right)}{{C}_{0}\left(\frac{\mu\:g}{ml}\right)}*\frac{1}{A\left({cm}^{2}\right)}*\frac{{C}_{t}\left(\frac{\mu\:g}{ml}\right)}{t\left(sec\right)}$$

V = volume receiving chamber. C_0_ = initial concentration of dextran in donor chamber. A = surface area of the membrane. C_t_ = concentration of dextran in the receiving chamber. t = time of incubation.

### Immunocytochemistry

Transwell inserts containing PODO/TERT256 used for the filtration assay were further imaged for F-actin. After removing the supernatant, cells were fixed with 4% PFA (para-Formaldehyde for 30 min at room temperature. After washing 3 times with PBS the membranes on the bottom were cut out with a scalpel, placed on an even surface of parafilm and incubated in Phalloidin iFluor488 (Cat# ab176753, Abcam, 1:1000 in 1% BSA/TBS-T) solution for 1 h at room temperature in the dark. After washing the membranes 3 times with PBS they were transferred onto glass slides, mounted using fluorescence mounting medium (Cat# S3023, Dako), covered with a glass coverslip and sealed with nail polish. Cells were imaged using a fluorescence microscope (EVOS M5000, Invitrogen). Images were adjusted for display using ImageJ.

### Impedance

PODO/TERT265 (Evercyte GmbH, Austria) were cultivated in DMEM/F12 (1:1) (Thermo Fisher Scientific, Waltham, USA) supplemented with GlutaMax (1%, Thermo Fisher Scientific), ITS-G (1%, Thermo Fisher Scientific), FBS (Bio&Sell), Pen/Strp (1%, Thermo Fisher Scientific) and medium was renewed every 2–3 d. PODO/TERT265 cells were seeded at a density of 25,000 cells/cm^2^ on Rat Tail Collagen I (50 µg/mL in PBS (w/o Ca, Mg); R&D Systems, Minneapolis, USA) coated E-Plates 96 (Agilent, Santa Clara, USA). Cells were cultivated for at least 7 d to ensure a stable impedance level before start of compound treatment. Baseline and post-treatment impedance were assessed using a xCELLigence Real-Time Cell Analysis (Agilent) device. IK-930, VT-107 and K-975 were dissolved in DMSO and treatment of cells with compounds was performed in medium containing 0.25% DMSO.

### ATP measurement

The ATP content of PODO/TERT256 cells was quantified using the ATP CellTiter-Glo (Promega) according to the manufacturers protocol. In brief, the CellTiter-Glo Reagent (substrate solubilized in buffer) was added to the cell culture medium and incubated for 2 min at 115 rpm on the plate rocker. Subsequently, cells were incubated for 10 min at room temperature in the dark for stabilization of the signal. Cell lysate was transferred to a white cell culture plate (Perkin Elmer, Shelton, USA) and luminescence was measured using the Spectra Max i3X (Molecular Devices, San Jose, USA).

### Seahorse extracellular flux assay

PODO/TERT256 cells were seeded in Seahorse XFe24 culture plates (Agilent) and cultured for 7 d. Cells were analysed using an adapted version of the Seahorse XF Cell Mito Stress Kit with TEAD inhibitors or DMSO being injected via port A. After 60 min coupling of mitochondria was assessed by addition of oligomycin A (1.5 µM) via port B, FCCP (1.5 µM) via Port C and subsequent addition of rotenone (0.5 µM) and antimycin A (0.5 µM) via port D in 18 min intervals. All concentrations indicated were final concentrations used. Oxygen consumption rate (OCR) and extracellular acidification rate (ECAR) values were exported from the Seahorse Wave Desktop Software (Agilent) to Excel. All values were displayed as percentage of the individual baseline before compound injection.

### Statistical analyses

Unless otherwise indicated data are presented as mean ± SEM. Sample size (n) indicates the number of independent replicate experiments performed. Statistical significance was determined using GraphPad Prism Version 8. Information on statistical tests employed are provided in the figure legends.

## Results

### TEAD inhibitors affect the viability of PODO/TERT256 cells

**Fig. 2:**
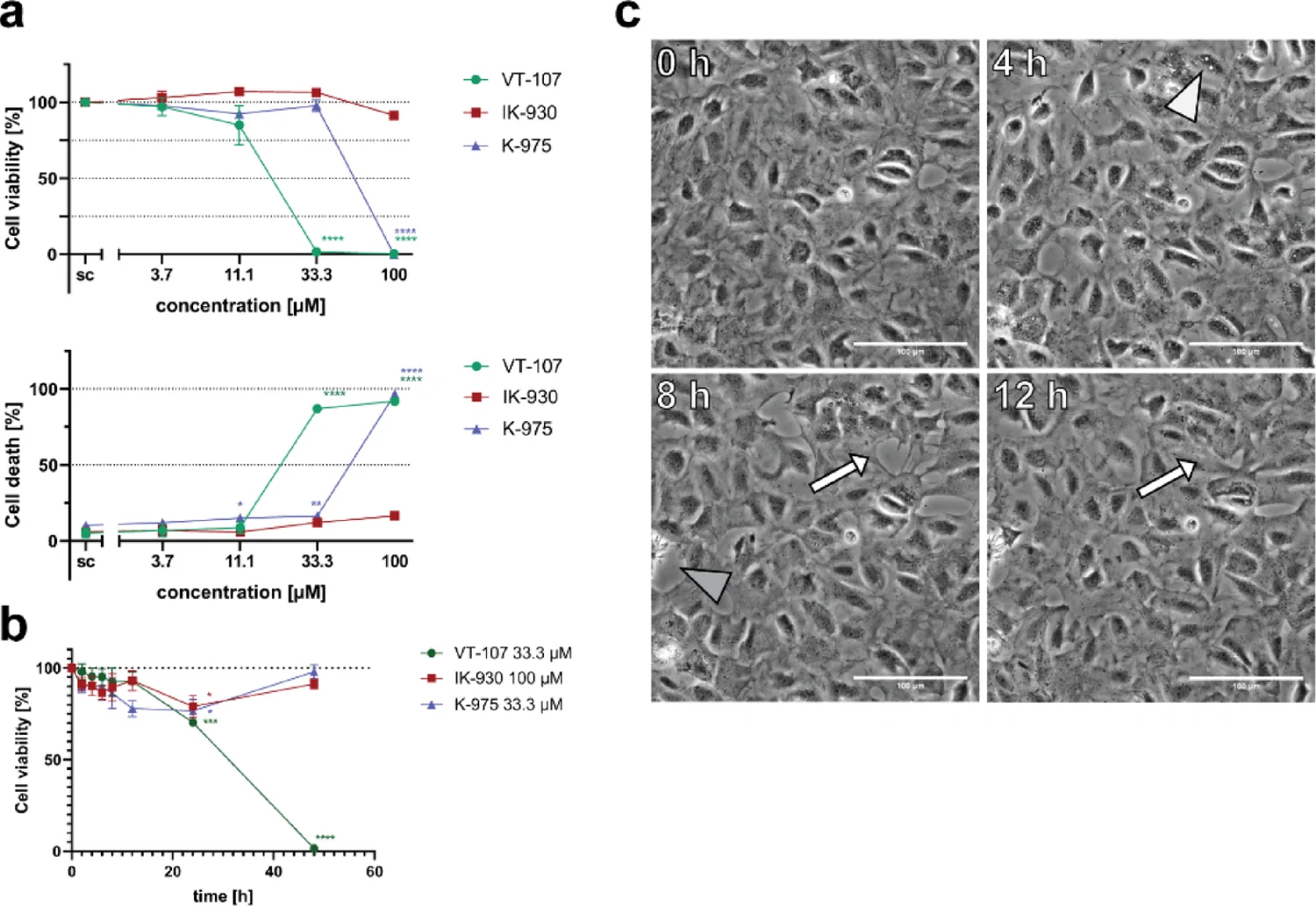
Viability and cell death of PODO/TERT256 after treatment with the TEAD inhibitors VT-107, IK-930 and K-975. Bright field microscopic images after IK-930 treatment. Cell viability and cell death assessed via resazurin reduction and LDH leakage after 48 h of TEAD inhibitor exposure (**a**). *n* = 3, mean ± SEM. Cell viability of PODO/TERT256 assessed after various time points of 33.3 µM VT-107, 100 µM IK-930 and 33.3 µM K-975 exposure (**b**). *n* = 3, mean ± SEM. One-way ANOVA followed by Dunnett’s multiple comparison test relative to the respective solvent control. Significant differences: * = *p* ≤ 0.05; ** = *p* ≤ 0.01; *** = *p* ≤ 0.001; **** = *p* ≤ 0.001. PODO/TERT256 grown in T25 flask for 1 week and treated with 50 µM IK-930 for 12 h (**c**). Scale bar = 100 μm, representative images. The white arrowhead (∇) indicates intracellular vesicles that appear after TEAD inhibitor treatment. The white arrow (↑) indicates the opening of the monolayer, while the grey arrowhead (∇) points to crystal structures

Since TEAD inhibitors exhibited nephrotoxicity, apparently specifically targeting the glomerulus (Paul et al. 2025), human immortalized podocytes were used to assess toxicity in vitro. Confluent PODO/TERT256 were exposed to up to 100 µM of the TEAD inhibitors VT-107, IK-930 and K-975 for 48 h. Despite targeting the same hydrophobic pocket, VT-107 showed the highest toxicity as 33.3 µM led to a significant decrease in cell viability. IK-930 did not evoke major cellular toxicity at concentrations ≤ 100 µM whereas K-975 led to a significant reduction of cell viability only at 100 µM (Fig. 2a). In addition, resazurin reduction was studied over the course of 48 h to analyse the compound effect on cell viability over time as well as potential recovery. Indeed, 100 µM IK-930 and 33.3 µM of K-975 decreased cell viability significantly at 24 h but no difference was observed at 48 h, meaning cells were able to recover even in the presence of compounds. VT-107 at 33.3 µM continuously decreased cell viability over time with no signs of recovery (Fig. 2b). To better characterize the recovery effect during IK-930 exposure, images of the cell monolayer were taken before and after IK-930 exposure. 4 h post treatment with 50 µM IK-930, cells were characterized by starting to retract from cell-cell contacts, appearance of intracellular vesicle-like structures, followed by random loss of cell-cell contact at 8 h, and cell enlargement and re-establishment of cell-cell contacts at 12 h (Fig. 2c). The latter, possibly suggesting that IK-930 was being actively sequestered into intracellular vesicles.

### TEAD inhibitors lead to acute morphological alterations

**Fig. 3:**
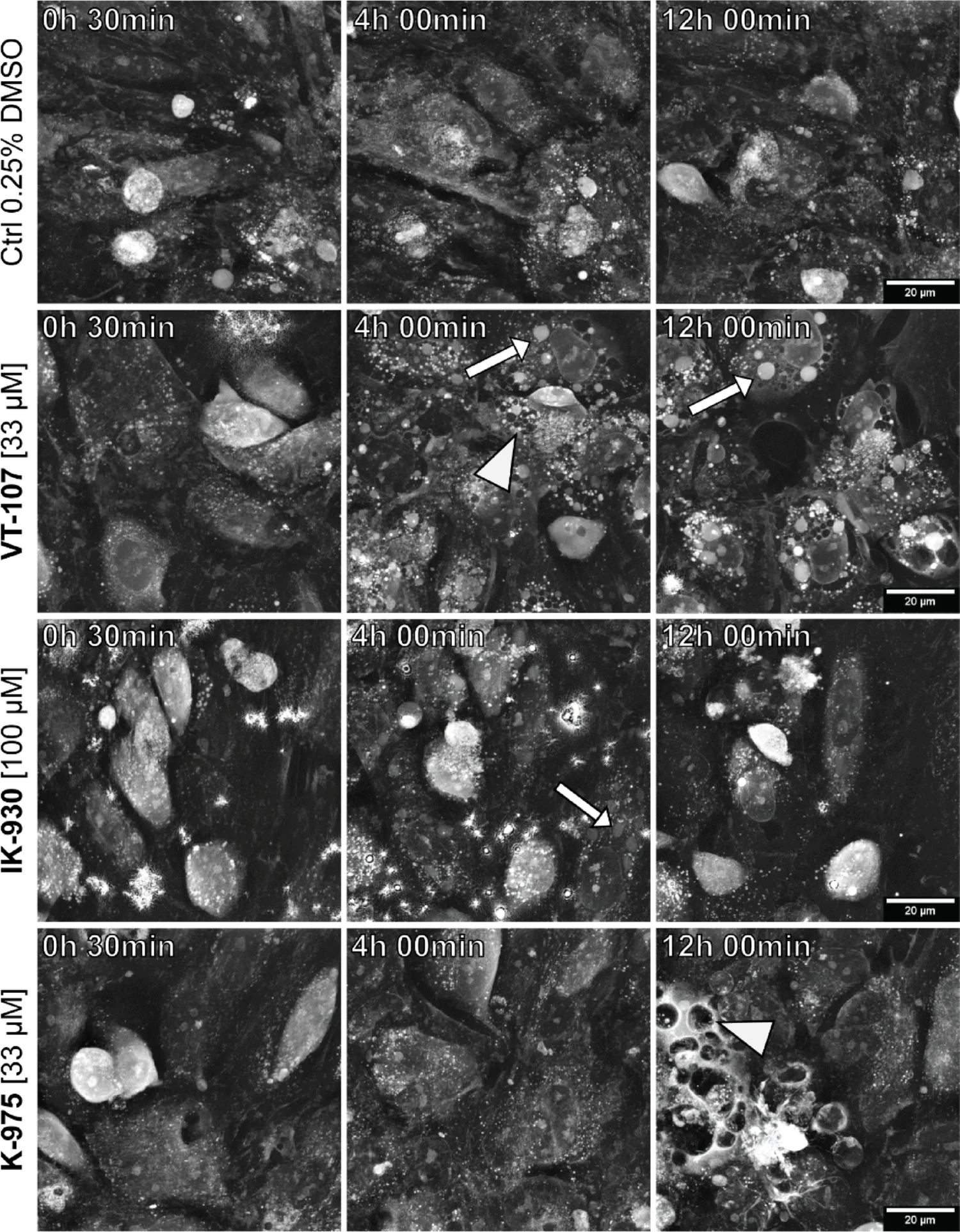
Holotomography images of PODO/TERT256 cultured for 2 d and either treated with 0.25% DMSO, 33.3 µM VT-107, 100-µM IK-930 or 33.3 µM K-975. The white arrow (↑) points at newly formed bright vesicles with a high refractory index. The white arrowhead (∇) highlights dark round areas with a low refractory index. Pictures were taken every 30 min and sections shown are representative of the whole 3 × 3 grid investigated. Scale bar = 20 μm

To investigate the effect of TEAD inhibitors on PODO/TERT256 cells over time in more detail cells were imaged using a NanoLive microscope over a 24 h exposure period. The holotomography obtained suggested the formation of light grey intracellular cytoplasmic vesicles upon treatment with 100 µM IK-930, 33.3 µM VT-107 and 33.3 µM K-975, already visible at 30 min following initial exposure. These vesicles continued to form over subsequent h, increasing in number and size, especially after VT-107 treatment. In contrast, in cells treated with IK-930, vesicle formation did not coincide with overt cell death, but rather recovery is observed. The latter observations confirm earlier findings with cell viability and microscopic characterization (Fig. 2). Exposure to VT-107 and IK-930 also led to the formation of dark round vesicles in the cytosol reminiscent of fluid filled macropinosomes (NanoLive 2019). These dark round vesicles can also be observed in cells treated with 33.3 µM K-975 after 12 h and coincided with cell death at 24 h (Fig. 3).

### TEAD inhibitors disrupt the glomerular filtration barrier

**Fig. 4:**
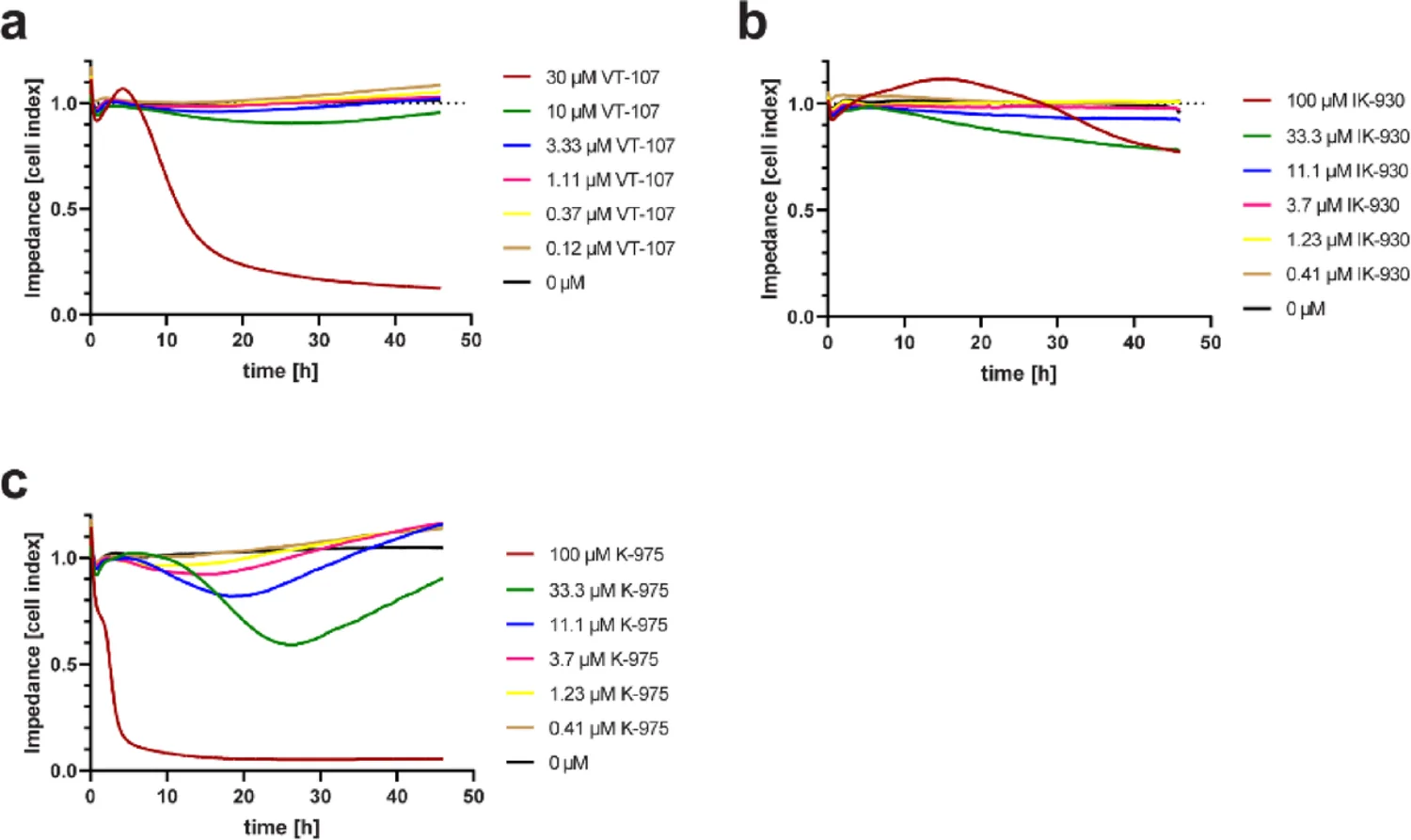
Concentration-dependant decrease of PODO/TERT256 monolayer impedance by TEAD inhibitors. PODO/TERT256 cells were cultivated for 7 d and treated with different concentrations of VT-107 (**a**), IK-930 (**b**) and K-975 (**c**) up to 30 µM, 100 µM and 100 µM, respectively. The change of impedance (cell index) during the treatment to its initial values was depicted on the Y-axis. *n* = 5 for VT-107, *n* = 2 for IK-930 and K-975

In conjunction with the observed morphological changes upon TEAD inhibitor exposure in PODO/TERT256 monolayers as well as the reported reversibility of protein/albuminuria in rodents, we aimed at characterizing the observed changes in cell size and cell contacts (Figs. 2 and 3) via determination of cellular impedance (Xu et al. 2016) in PODO/TERT256 upon treatment to demonstrate changes in cell and barrier integrity. Indeed, all compounds induced dose-dependent alterations in impedance (Fig. 4) already at concentration ranges that were not cytotoxic (Fig. 2). However, the change in impedance differed amongst TEAD inhibitors. VT-107 induced a minor concentration-dependent drop at concentrations ≤ 10 µM, with overt cytotoxicity at 30 µM (Fig. 4a). Similar to VT-107, IK-930 demonstrated a clear concentration-dependent drop in impedance (Fig. 4b), albeit the change in impedance at 33.3 µM was much more gradual than the one observed for VT-107 at 30 µM, commensurate with the limited cytotoxicity observed for IK-930 at 33.3 µM (Fig. 2b). K-975 at concentrations ≤ 33.3 µM induced a drop in impedance after single dose treatment with nearly complete recovery at 48 h (Fig. 4c). More importantly, these changes in impedance were already observed at non-cytotoxic concentrations (3.7, 11.1, and 33.3 µM).

**Fig. 5:**
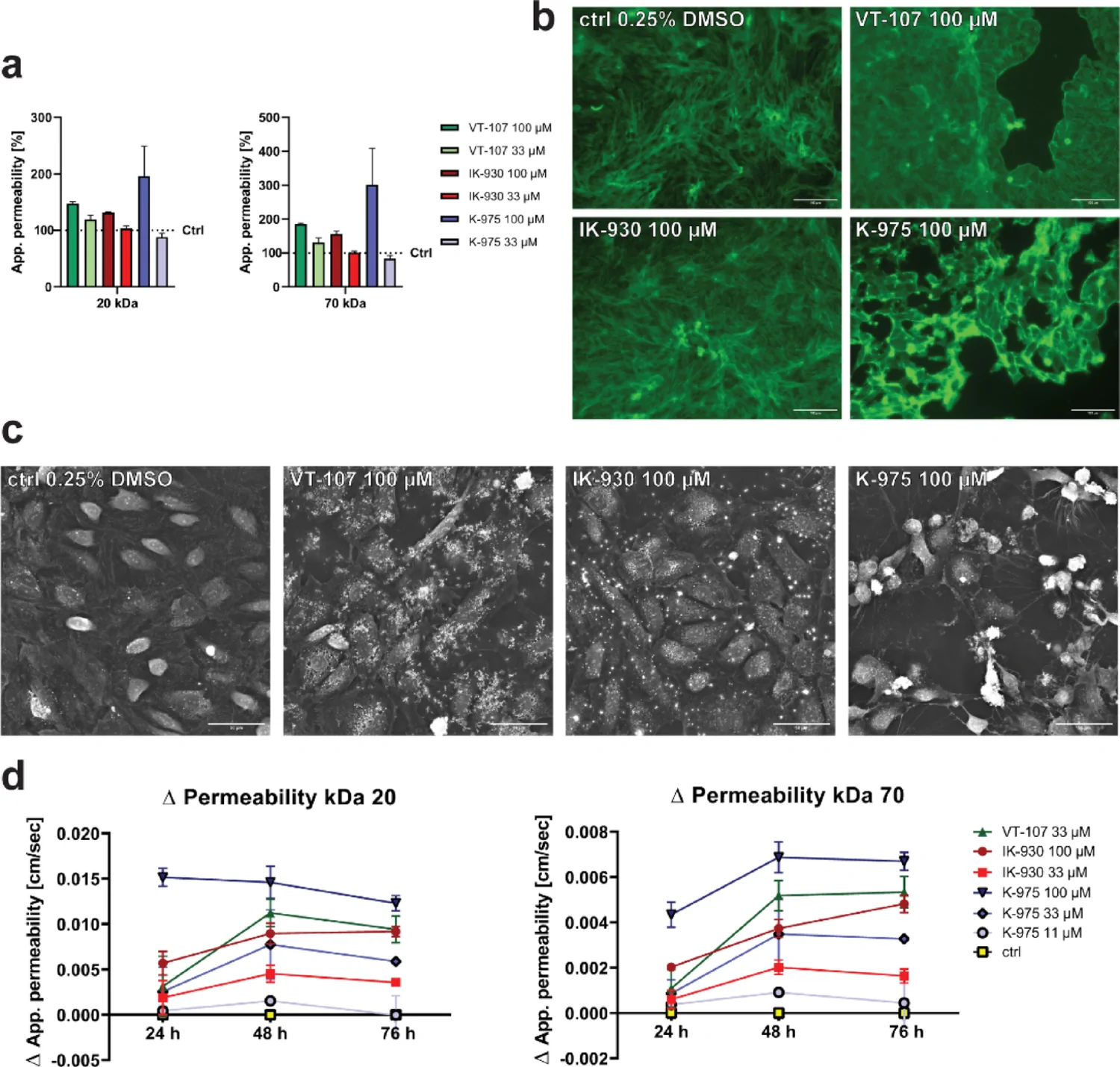
Apparent permeability and overview images of PODO/TERT256 monolayers. Apparent permeability for two fluorescently labelled dextrans (20 and 70 kDa) after 4 h of TEAD inhibitor exposure (**a**). PODO/TERT256 stained for F-actin after 4 h of TEAD inhibitor exposure (**b**), representative images. Scale bar = 100 μm. Holotomography pictures of PODO/TERT256 cells 3 h and 50 min after treatment of 100 µM VT-107, IK-930 and K-975 (**c**). Scale bar = 50 μm. Apparent permeability after 24 h, 48 h and 76 h of TEAD inhibitor exposure when compared to control (**d**). *n* = 3, mean ± SEM

To confirm above changes in cell morphology and electrophysiology, which could affect monolayer integrity, the monolayer function and filtration capacity of PODO/TERT256 cells was investigated by testing the size dependent permeability of the monolayer using 20 kDa and 70 kDa fluorophores in transwell assays over 4 h. The apparent permeability increased for all TEAD inhibitors tested at their maximum concentrations (Fig. 5A). However, while VT-107 and K-975 were shown to be cytotoxic at 100 µM, this was not the case for IK-930 (Fig. 2). Obviously, cytotoxicity and thus destruction of the cell monolayer would need to result in excess apparent permeability of both the 20 kDa and 70 kDa fluorophores. Indeed, F-actin staining of PODO/TERT256 cells after the 4 h TEAD inhibitor treatment confirmed the rapid onset of cell death at 100 µM K-975, in comparison to 100 µM VT-107 (Fig. 5b), which support data from the time course experiment (Fig. 2b). While the latter also holds true for VT-107 at 33 µM, neither IK-930 nor K-975 were cytotoxic at these concentrations and thus also did not demonstrate an increased apparent permeability, despite observable changes in impedance (Fig. 4). Holotomography from an additional experiment showed a similar outcome (Fig. 5c), as 100 µM of VT-107 and IK-930 led to cells retracting from surrounding cells, enabling more fluorophores to pass the monolayer, but not to massive and rapid cell death as evoked by K-975. Already at 30 min of exposure, 100 µM K-975 led to visible cell death (data not shown). Thus K-975 affected PODO/TERT256 cells rapidly, whilst VT-107 acted in a continuous manner, which was confirmed by the analysis of permeability over the course of 3 d (Fig. 5d). It is interesting to note that non-cytotoxic concentrations of 100 µM IK-930 and 33 µM K-975 led to increased apparent permeability of 70 kDa fluorophores (Fig. 5c), similar to the observed changes in impedance (Fig. 4), thereby suggesting that TEAD inhibitors were able to decrease the effectiveness of the filtration barrier also at non-cytotoxic concentrations.

### Impact on energy metabolism of TEAD inhibitors

**Fig. 6:**
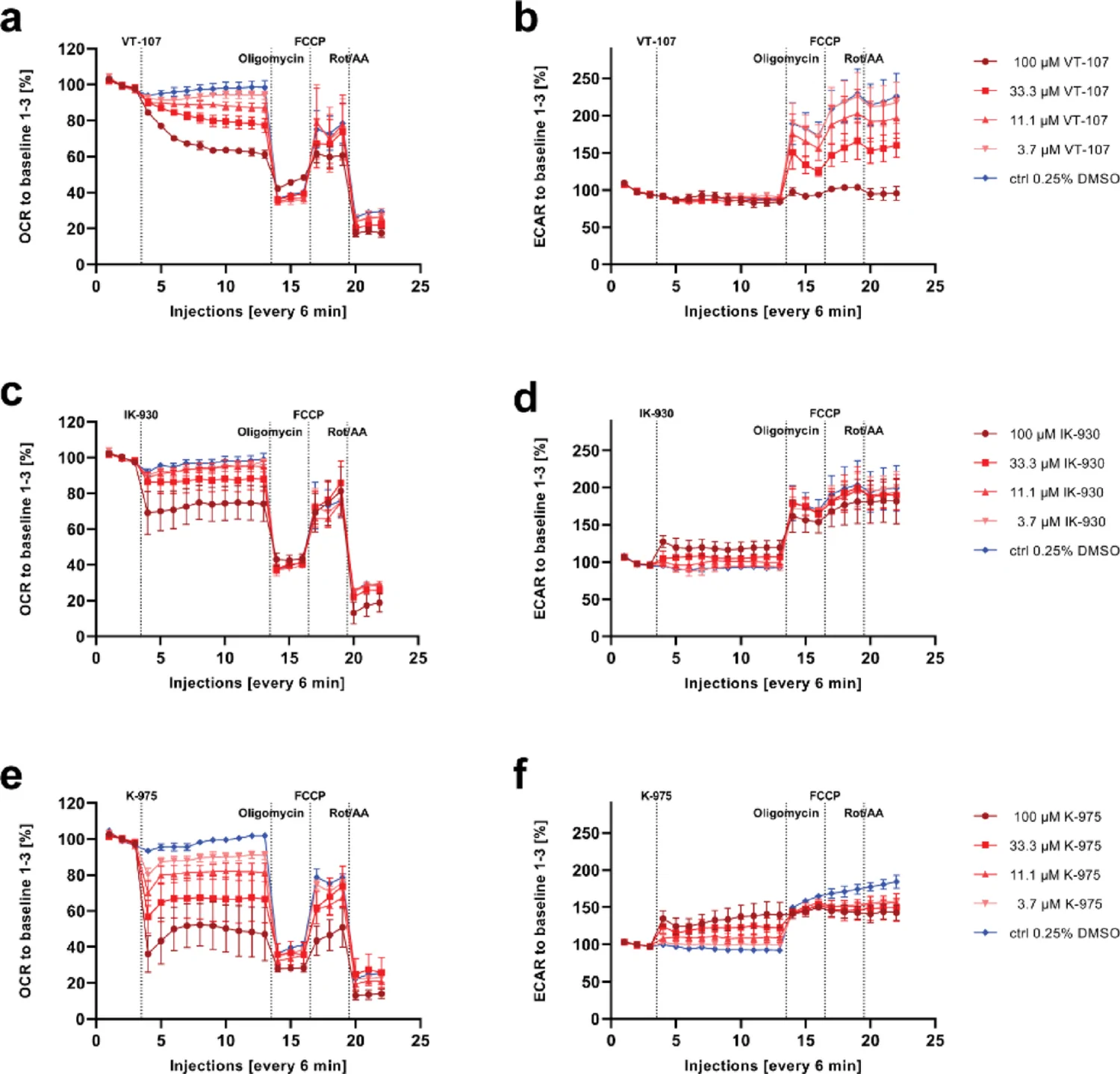
Metabolic effect of TEAD inhibitors on PODO/TERT256. The oxygen consumption rate (OCR) and extracellular acidification rate (ECAR) were measured after injection of various concentration of VT-107 (**a**, **b**), IK-930 (**c**, **d**) and K-975 (**e**, **f**) over the course of 1 h. *n* = 3, mean ± SEM

As podocytes rely on ATP to remodel their filtration slit and cytoskeleton, which is essential for proper filtration function of the glomerulus (Takahashi et al. 2024), the effect of TEAD inhibitors on PODO/TERT256 metabolism was characterized using the Seahorse analyser. Oxygen consumption rate (OCR) and extracellular acidification rate (ECAR) were measured upon IK-930, VT-107 and K-975 acute injection. Overall, all three TEAD inhibitors induced a concentration-dependant decrease in OCR and concurrently an increase of ECAR. VT-107 led to a decrease in OCR over the course of 30 min, reaching 65% at 100 µM with no major compensation by glycolysis as typically indicated by elevated ECAR levels (Fig. 6a, b). In contrast injection of IK-930 induced an immediate concentration-dependent decrease in OCR and a concomitant, however, moderate concentration dependent increase in ECAR (Fig. 6c, d). K-975 affected cells similarly, however to a larger extent, meaning K-975 appeared to be more potent in impacting energy metabolism (Fig. 6e, f). Overall, TEAD inhibitors not only affected mitochondrial oxidative phosphorylation but also compensatory glycolysis in a concentration dependent manner, thus corroborating the earlier observation of reduced cytoskeletal reorganization due to TEAD inhibitor induced changes in ATP generation and availability.

### Repeated dosing elevate PODO/TERT256 sensitivity to TEAD inhibitors

**Fig. 7:**
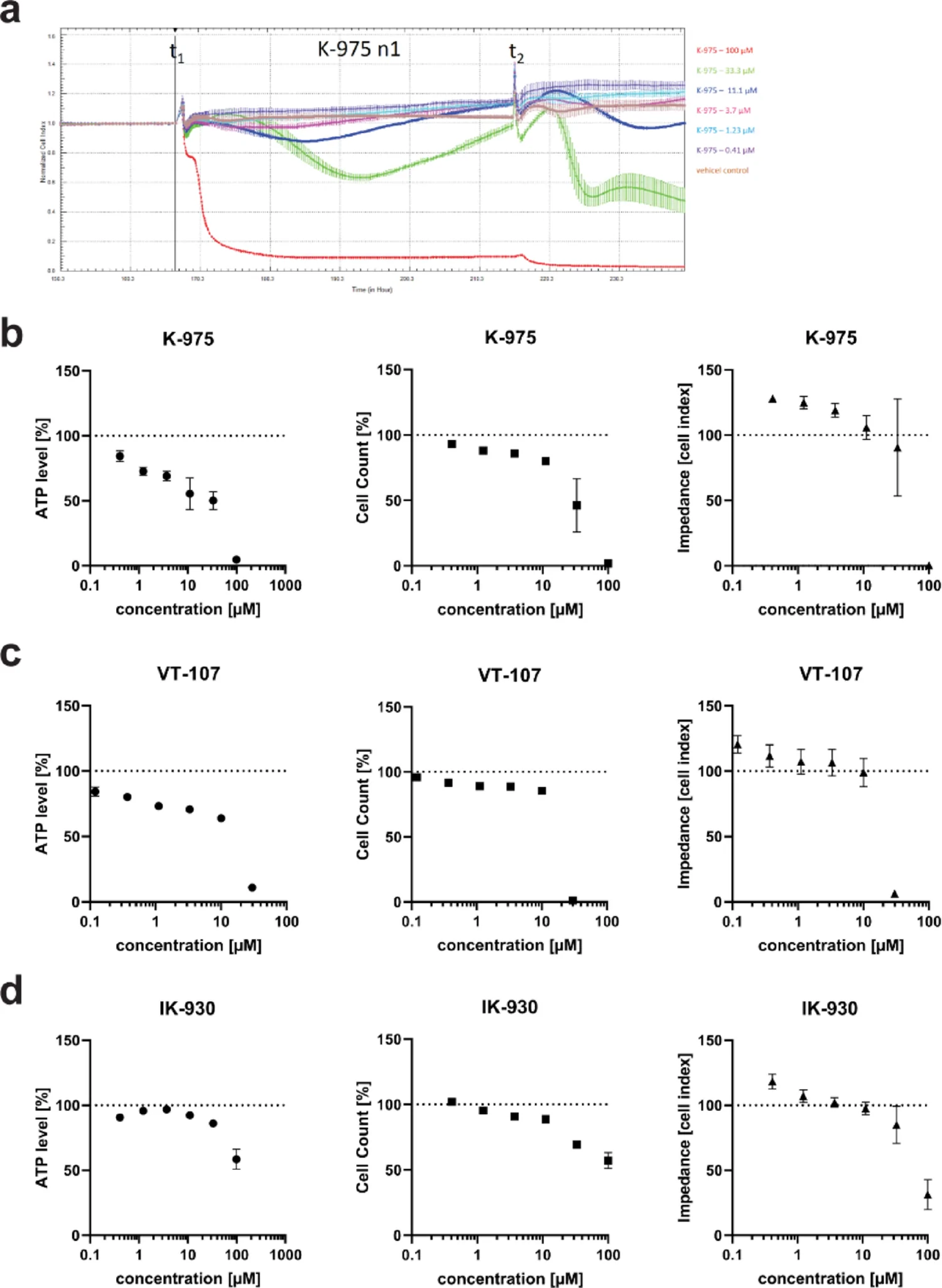
ATP Level, Cell count and impedance after repeated dosing with TEAD inhibitors. PODO/TERT256 cells were cultured for 7 d and treated with TEAD inhibitors once (t_1_) and again after 48 h (t_2_). The first replicate of the impedance measurement over the treatment period is displayed (**a**). *n* = 1, 3 technical replicates, mean ± SD. After the 72 h period cellular ATP level, cell count and impedance was determined for different concentrations of K-975 (**b**), VT-107 (**c**) and IK-930 (**d**). *n* = 2 for IK-930 and K-975, *n* = 5 for VT-107, mean ± SEM

To determine whether repeated exposure to TEAD inhibitors could lead to increased toxicity, as was suggested by in vivo studies (Otsuki et al. 2024), PODO/TERT256 cells were treated with TEAD inhibitors repeatedly, i.e. at 0 and at 48 h. Non-cytotoxic 33 µM K-975 induced a drop of impedance to 60% after the first treatment, with an almost complete recovery until the second treatment at 48 h. The 2nd treatment lowered impedance to 50% and no recovery was observed at 72 h (Fig. 7a). Indeed, at 72 h K-975 induced cell death as demonstrated by a decreased cell count and lowered impedance values. It is noteworthy that ATP levels were lowered already at much lower K-975 concentrations, before changes in cell count and impedance were observed, suggesting a dramatic effect of K-975 on energy metabolism (Fig. 7b). VT-107, similar to K-975, led to a drop in ATP content before cell toxicity became apparent in concentration ranges of 1–10 µM (Fig. 7c), aligning well with the Seahorse analyser data (Fig. 6a). In contrast, repeated dosing with IK-930 did not demonstrate this decrease in ATP level, cell viability or impedance with the exception of when 100 µM were employed (Fig. 7d), possibly suggestive of a compensatory reaction.

### Effect of repeated dosing in combination with palmitic acid

**Fig. 8:**
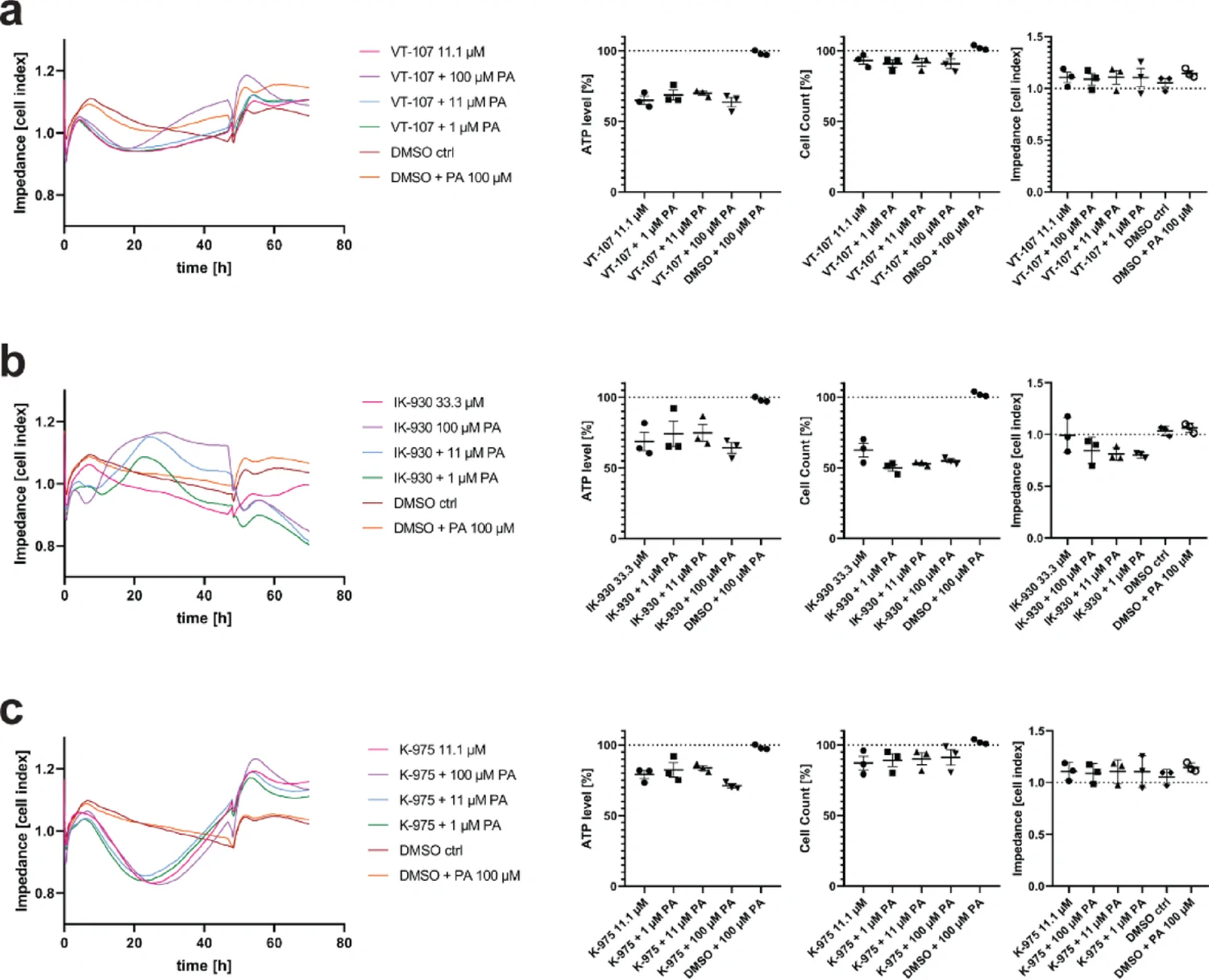
ATP level, cell count and impedance after 72 h of TEAD inhibitor treatment. PODO/TERT256 cells were cultured for 7 d and treated with VT-107 (**a**), IK-930 (**b**) and K-975 (**c**) for 48 h and a second time for 24 h. Pictures on the left display the impedance over the course of the treatment. After 72 h, impedance was recorded a final time at which ATP levels and cell counts were measured. *n* = 3, mean ± SEM

K-975, IK-930 and VT-107 bind to the S-palmitoylation site inside the hydrophobic pocket of TEAD blocking TEAD-Yap/TAZ interaction. Accordingly, we assumed that excess concentrations of palmitic acid should elevate palmitoyl-Coa levels and thus modulate the effect of TEAD inhibitors in PODO/TERT256 cells if the adverse effects of TEAD inhibitors are causally related to the TEAD blocking of the TEAD-Yap/TAZ interaction. However, treating cells with 11.1 µM VT-107 and either 1, 11 or 100 µM palmitic acid (PA) did not change ATP level, cell count or impedance after 72 h of exposure. At 100 µM PA and 11.1 µM VT-107 impedance increased faster at the 20 h mark (Fig. 8a), but had no effect on either ATP levels, or cell viability. Co-exposure with palmitic acid and 33.3 µM IK-930 greatly affected impedance during the first 24 h, whereas again no effect was observed with regard to ATP levels or cell viability. Not only impedance increased at the 20-h mark in a concentration-dependent manner, but impedance measurements also demonstrated a biphasic curve initially decreasing during the first h before increasing even above corresponding control levels at 48 h (Fig. 8b). In contrast, palmitic acid co-exposure had no effect at all in PODO/TERT256 cells treated with 11.1 µM K-975 (Fig. 8c).

## Discussion

Treatment-resistant metastatic tumors, including solid tumors and mesotheliomas, often exhibit poor prognostic outcomes due to alterations in the Hippo signalling pathway (Harvey et al. 2013; Nguyen and Yi 2019). Recent therapeutic strategies have therefore focused on preventing the interaction between TEAD transcription factors and their coactivators YAP/TAZ, the last step in the Hippo pathway, thereby limiting uncontrolled transcription of proliferation and migration genes (Cunningham and Hansen 2022). In addition to approaches that reduce nuclear TEAD levels through PROTAC-mediated degradation (Chen et al. 2024; Li et al. 2024) or directly disrupt protein–protein interactions by targeting interface regions (Chapeau et al. 2024) of the TEAD protein, small molecules preventing the palmitoylation of the hydrophobic central pocket have emerged. The largely inhibited palmitoylation of the central pocket conserved in all 4 TEAD isoforms leads to the destabilization of the TEAD-YAP/TAZ binding and has shown promise as a therapeutic strategy (Pobbati et al. 2015). However, treatment-associated adverse effects such as albuminuria caused by renal injury have been reported during preclinical and clinical evaluation (Kaneda et al. 2020; Yap et al. 2023a; Young et al. 2023). In conjunction with known species specific effects (Chu et al. 2013), the latter findings thus emphasized that more in-depth testing of new drug candidates in preclinical studies is highly warranted. These additional studies should not be limited to animal testing, but more specifically also include appropriate and relevant in vitro models (Leist and Hartung 2013; McKim Jr 2010; Olson et al. 2000) with human cells.

In the quest for a more appropriate in vitro model for TEAD induced adverse effects such as albuminuria and renal injury, we aimed to characterize the impact of the TEAD 1 inhibitor IK-930 (Tolcher et al. 2022; Young et al. 2023) and the two pan-TEAD inhibitors VT-107 and K-975 (Kaneda et al. 2020; Tang et al. 2021) on podocytes via an in vitro model system consisting of human immortalized PODO/TERT256 cells (Schlichenmaier et al. 2024). Accordingly, the evaluation of TEAD-inhibitor induced changes in cell death at 48 h of exposure demonstrated significant effects as of 11.1 µM VT-107, 33.3 µM K-975 but only a slight but non-significant increase in cell death at 100 µM IK-930 (Fig. 2). The latter emphasizing the stark differences of human podocytes in sensitivity to the three TEAD inhibitors tested. More importantly, treatment with 33.3 µM IK-930 and 33 µM K-975 over the 48-h time-course was characterized by a transient decrease in viability (resazurin) up to 24 h of exposure and followed by partial or full recovery over the next 24 h, suggesting some form of an adaptive response missing for VT-107. Indeed, repeated exposure experiments appeared to corroborate the latter findings (Fig. 7), as little change was observed in the IK-930 treatment, whereas for K-975 the first treatment resulted in a recovery of the cells at 48 h, but the 2nd exposure resulted in loss of ATP and cell viability. As noted earlier, no adaptive response was observed with repeated exposure to VT-107, where cell viability reached 0% at 72 h. As these findings were also supported by a comparable exposure response pattern, when determining permeability of the monolayer to fluorescent dextrans (Fig. 5), changes in impedance (Fig. 4) and ATP levels (Figs. 6 and 7), TEAD-inhibitor dependent induced morphological alterations, including cell death affecting the cell monolayer must have occurred.

All three TEAD-inhibitors induced the formation of vacuoles (dark round structures), which were presumed to represent macropinosomes. In addition, VT-107 and IK-930 led to the formation of vesicles (light grey structures) that fused over time. While podocytes were reported to conduct macropinocytosis to maintain the filtration barrier (Tian et al. 2022), the excessive formation of vacuoles and vesicles suggested either deregulated vesicle and vacuole formation (Shubin et al. 2016) and/or a lack of cellular energy (Zhang and Commisso 2019). In the data presented here, major vacuole formation was often correlated with cell death, initial cell shrinkage and retraction at 33.3 µM IK-930, followed by subsequent modest restoration of the monolayer structure (Fig. 2), which was absent for VT-107 and K-975 at comparable concentrations. Whether or not, the highly conserved pathways of micropinocytosis (Tu et al. 2025) served to sequester crystallized and/or aggregated TEAD-inhibitor compounds as would be suggested for IK-930 (Fig. 2), only vesicle content specific analyses would allow confirmation. As the process of micropinocytosis (Tu et al. 2025) is initiated by localized ATP demanding actin polymerization (Baek et al. 2023; Takahashi et al. 2024), which drives the formation of membrane protrusions that close to generate macropinosomes, any change in metabolism that decreases in ATP generation would most likely result in a changed rate of micropinocytosis. Thus, podocytes exposed to highly cytotoxic TEAD-inhibitors, e.g. VT-107 (Fig. 2) that also affect ATP generation (Fig. 6a, b), would unlikely be highly successful in reducing cell exposure via engulfing VT-107 via micropinocytosis. In contrast, less cytotoxic but clearly ATP decreasing TEAD-inhibitors, e.g. K-975 (Figs. 2 and 6), would, at least after a single exposure, be able to initiate micropinocytosis, possibly sequestering K-975, but commensurate with the increased inhibition of ATP generation upon a 2nd exposure, succumb to overall cytotoxicity. In stark contrast to the latter, IK-930 with some reduction in ATP generation at the very highest concentrations, demonstrated crystalline compound deposits in the media and vesicles possibly containing compound and consequently marginal cytotoxicity. Overall, live cell imaging (Fig. 3) demonstrated that during the first hour of exposure cell-cell contacts dissolved and the surface spanning podocytes appeared to recede to the point of rounded-up cell bodies. The latter separation of cells is a known stress response (Shirato 2002) and thus could explain the TEAD inhibitor mediated effect on the filtration capabilities of the kidney (Meliambro et al. 2017).

Intrigued by the differences in response dynamics and morphological alterations induced by the three TEAD inhibitors, we next sought to characterize their impact on ATP generation, as the continuous remodelling of foot processes and maintenance of the filtration barrier are highly ATP-dependent (Baek et al. 2023; Takahashi et al. 2024). As already indicated above, IK-930, K-975 and VT-107 induced an immediate concentration-dependent decline in oxygen consumption rate (OCR) subsequent to injection (Fig. 6), suggesting rapid suppression of mitochondrial oxidative phosphorylation (OXPHOS). Indeed, TEAD1 deficiency in mouse renal tubular epithelial cells resulted in a significant decline of OCR after cisplatin treatment (Tran et al. 2025), while other groups showed that TEAD1 knock-out in cardiomyocytes led to reduced OXPHOS capacity (Liu et al. 2020). Consistent with the latter, a compensatory increase in extracellular acidification rate (ECAR) was observed after exposure to IK-930 and K-975, reflecting enhanced glycolytic activity to offset the loss of ATP generation by OXPHOS and has been shown to occur in other kidney in vitro systems (Secker et al. 2018). Notably, VT-107 did not induce this compensatory ECAR increase and, in a concentration-dependent manner, attenuated the typical metabolic responses to the OXPHOS modulators oligomycin, FCCP and rotenone (Heinz et al. 2017; Maro et al. 1982; Shchepina et al. 2002). Although the kinetic appeared to slightly differ amongst compounds, as VT-107 induced a gradual reduction of OCR reaching a plateau after 30 min, the immediate decline in OCR was unexpected. Effects driven by the transcriptionally regulated Hippo pathway are thought to induce a later onset of these metabolic changes. The latter led to the hypothesis of a possible off-target driven effect, e.g. compounds interfering with other cellular palmitoylation processes as there are more than 100 palmitoylation sites known (Collins et al. 2017) and mitochondrial energy generation is dependent on palmitoylation (Kathayat et al. 2018; Shen et al. 2017). As the pan-inhibitors VT-107 and K-975 reduced the ATP-levels at non-cytotoxic concentrations, while not affecting cell counts and impedance (Fig. 7), the short term effects on OCR and ECAR suggested impaired ATP generation over long term treatment that could impede proper kidney function and eventually drive disruption of the glomerular filtration barrier (Brinkkoetter et al. 2019; Imasawa and Rossignol 2013). Moreover, the addition of palmitate led to a faster recovery after exposure to VT-107 and IK-930 and confirmed the reported presumed protective effect of palmitate against VT-104 induced inhibition of proliferation in NCI-H226 cells (Nutsch et al. 2025). This suggested protective effect, however, ultimately did not alleviate the effects of the three TEAD inhibitors on cell count and ATP level after 72 h of repeated exposure (Fig. 8).

Above differences of IK-930, VT-107 and K-975 in vitro toxicity characteristics corroborated earlier findings for renal toxicity in preclinical animal models (Young et al. 2023), clinical trials (Paul et al. 2025) and in vitro cytotoxicity tests (Cunningham et al. 2025), thereby emphasizing the suitability of our human podocyte test system employed. The commonly reported side effect of proteinuria is in line with the concentration-dependent decrease in impedance, a surrogate of morphological alteration (Fig. 3), and an increase in permeability (Fig. 5). The observed effects on podocytes, and thus glomerular functionality, were noted in concentration ranges that did not induce cell death. The intensity of this impairment of functionality, as observed in the in vitro studies, may be an indication of the potential reversibility of this effect and thus of the proteinuria observed in vivo. Indeed, Otsuki et al. also observed recovery from albuminuria in rats after discontinuation of a daily dose of 300 mg/kg K-975 (Otsuki et al. 2024), albeit, some rats demonstrated only partial recovery. The promising pan-TEAD inhibitor VT3989, recently acquiring fast track designation by the FDA (Vivace Therapeutics 2025), has also been described to cause dose-related proteinuric nephropathy in animal studies (Yap et al. 2023b) and reversible proteinuria in a clinical trial of Phase 1/2 (Harvey and Tang 2025). Thus, inhibition of specific TEAD-isoforms may be beneficial due to reduced adverse side effects, however the efficacy has also been reported to be compromised, limiting their therapeutic potential (Paul et al. 2025) as was reported for IK-930.

In summary we hereby present the bridge between in vivo testing and our already established in vitro glomerular filtration barrier model. With the example of the TEAD inhibitors IK-930, VT-107 and K-975 we could recapitulate and better characterize the differences observed in vivo between potent pan-TEAD inhibitors and selective isoform inhibitors and potential recovery effects. In addition, the use of this in vitro system revealed an immediate inhibition of mitochondrial function by TEAD inhibitor exposure. Combining multiple endpoints enabled us to assess toxicological and metabolic glomerular effects of the compounds already at an early preclinical stage, as nephrotoxicity often is observed only in late clinical stages or even once a drug has entered the market (Connor et al. 2024; Tiong et al. 2014) Therefore, close monitoring of kidney function and the early onset of deteriorating filtration capacity is of high importance.

## Data Availability

No datasets were generated or analysed during the current study.
